# Microstructure and Property Modification of High-Strength Martensitic Steel Through Plasma Arc Remelting

**DOI:** 10.3390/ma19091908

**Published:** 2026-05-06

**Authors:** Yan Liu, Shilian Hu, Jianwen Huang, Bo Cai, Wenjuan Lei, Jun Hu, Yichao Wang, Yashan Guo, Han Wu, Huichuan Duan, Yongzhi Shi, Rui Jiang, Ruolan Wang, Jianxun Zhang

**Affiliations:** 1 Inner Mongolia Metal Material Research Institute, Baotou 014000, China; 2 Northern Materials Science and Engineering Research Institute, Ningbo 315103, Chinacatherinelwj@163.com (W.L.); glory.way@hotmail.com (Y.W.); gys9115@126.com (Y.G.); 18361824022@163.com (H.W.); duanhuichuan129@163.com (H.D.); yzshi1126@163.com (Y.S.); w924234533@163.com (R.W.); 3 State Key Laboratory for Mechanical Behavior of Materials, Xi’an Jiaotong University, Xi’an 710049, China; jxzhang@mail.xjtu.edu.cn

**Keywords:** martensitic stainless steel, plasma arc remelting, wear resistance, corrosion resistance

## Abstract

The microstructure of high-strength martensitic steel specifically made for additive manufacturing was modified via in situ plasma arc remelting (PAR) to improve its surface properties. The results reveal that the microstructure is characterized by the intragranular martensite and intergranular eutectic structure of high-strength martensitic steel. The intragranular worm-like δ-ferrite embedding in the martensite matrix was clearly observed after PAR. Compared with the as-deposited part, the tensile strength of the PAR part reached 1753 MPa, and the ductility increased to 2.3%. The strength and elongation had increased by 20% and 229%, respectively. After in situ PAR, the wear loss decreased to 80% of the tailored high-strength martensitic steel, and the corrosion current density decreased to 17%. Both the as-deposited part and the PAR part exhibited significant intergranular corrosion morphological characteristics.

## 1. Introduction

Martensitic stainless steels are a class of Fe-Cr-C alloys renowned for their high strength, hardness, and moderate corrosion resistance, making them critical structural materials in aerospace [1], heavy machinery, and ocean engineering [1,2]. The performance of these steels is intrinsically governed by their microstructure, which typically consists of a martensitic matrix and various secondary phases, primarily carbides [3].

The correlation between microstructure and mechanical properties is well established: Wear resistance in these alloys is predominantly controlled by the volume fraction, type, and distribution of hard intergranular eutectic carbides (such as M_7_C_3_) [4].

Despite these general principles, the wear and corrosion damage of parts [5] often occur randomly and suddenly in harsh service environments [6,7]. Use of remanufacturing technology, particularly surface modification, is one of the most efficient ways to recycle damaged parts and extend their service life [8,9]. However, conventional austenitic and ferritic stainless steels often fail to simultaneously meet the stringent requirements for both the high wear resistance and corrosion resistance demanded in heavy-duty applications [10,11,12]. Among martensitic stainless steels, AISI 431 (a high chromium Fe-Cr-C alloy) is one of the most widely used due to its balance of properties.

However, a critical scientific issue arises when adapting these alloys for additive manufacturing. Although high-chromium Fe-Cr-C alloys are typically designed for conventional processing routes such as casting and forging [10,13], their direct application in additive manufacturing often results in suboptimal mechanical properties.

**Figure 1:**
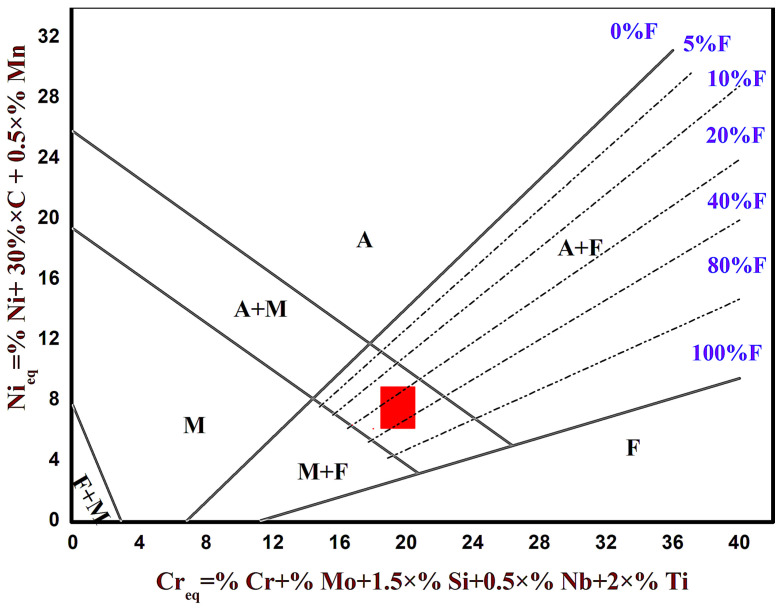
Designing martensitic stainless steel specifically for additive manufacturing based on Schaeffler’s diagram.

To address this challenge, a strategic modification of the alloy composition and processing methodology is required. Based on the compositions of AISI 431 stainless steel, a tailored 12Cr17Ni2B martensitic stainless steel powder was developed specifically for additive manufacturing [14]. This novel alloy incorporates boron (B) and vanadium (V) atoms, where the addition of B is beneficial for the formation of intergranular eutectic carbides to improve wear resistance [15], and the addition of V contributes to grain refinement [16,17]. Furthermore, as illustrated in Figure 1, Schaeffler diagram analysis reveals that the 12Cr17Ni2B alloy is situated in a mixed region of martensite (M), austenite (A), and ferrite (F). This specific microstructural design presents the feasibility of modifying the surface microstructure and properties of the parts through post-deposition treatment.

As shown in previous work by Liu et al. [18], the tailored 12Cr17Ni2B martensitic stainless steel powder was utilized to prepare the additive manufactured part with uniform mechanical properties. However, the resulting parts suffered from extremely limited ductility, with elongation values as low as 0.6%. This severe brittleness represents a significant bottleneck that restricts the application of additively manufactured martensitic steel in load-bearing components.

Additive manufacturing (AM) processes often result in anisotropic microstructures due to high thermal gradients and rapid solidification [19,20,21]. This anisotropy poses a challenge to the structural integrity of the parts. To address this limitation, this study employed in situ plasma arc remelting (PAR) to modify the surface microstructure. The secondary remelting process moderated the thermal gradients, transforming the columnar grains into a refined, equiaxed structure and eliminating the mechanical anisotropy.

The plasma arc cladding method is characterized by its efficiency potential, good densification, and cost-savings [22,23,24], which have shown great advantages in manufacturing [25] and part remelting [26] compared to laser cladding technology. Plasma arc cladding and in situ plasma arc remelting (PAR) technology has provided new methods for extending the service life of damaged parts through surface property modification in the field environment.

In this study, plasma arc remelting technology was utilized to regulate the wear resistance and corrosion resistance of the surface of the additively manufactured martensitic steel part.

## 2. Experimental Work

### 2.1. Materials and Methods

The chemical composition (wt%) of tailored 12Cr17Ni2B steel is shown in Table 1.

**Figure 2:**
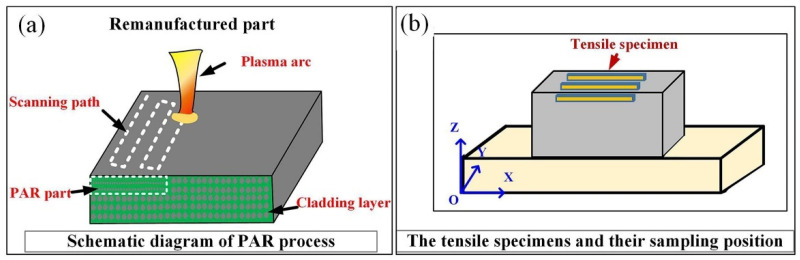
Schematics of (**a**) PAR treatment and (**b**) the sampling location for the tensile specimen of the as-deposited additive manufacturing parts and remelted part.

Constant-pressure DML-V03BD plasma arc cladding equipment (sourced from Beijing DML Technology Co., Ltd., Beijing, China) and a YASKAWA robot system (supplied by Yaskawa Electric Corporation, Kitakyushu, Japan) were used for in situ remelting on the surface of the part. A plasma arc cladding gun was employed to perform in situ PAR on the surface of the additive manufacturing parts. During the deposition process, the highly compressed and well-focused plasma arc remelted the surface of the as-deposited additive manufacturing parts according to the two-dimensional plane information of the slices. The travel path of the plasma arc cladding gun is shown in Figure 2a. The sampling positions of the tensile test specimens of the remelted parts are shown in Figure 2b.

**Figure 3:**
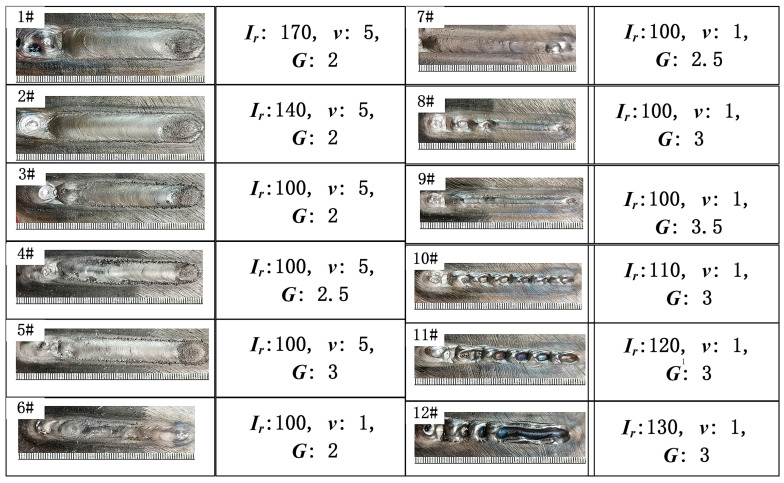
The macroscopic morphology of the PAR tracks.

As shown in Figure 3, processes 1# to 5# were obtained by changing the remelting current *Ir* (A) and ion gas *G* (L·min^−1^) parameters while keeping the powder feeding rate *v* (cm·min^−1^) at 5 r·min^−1^. The macroscopic morphology characteristics of the PAR tracks are shown in Figure 3.

It can be observed that a remelting current of 100 A was sufficient to remelt the surface metal of the as-deposited parts. As shown in Figure 3, processes 6# to 12# were obtained by changing the remelting current (*I_r_*) and ion gas (*G*) parameters while keeping the powder feeding rate at 1 r·min^−1^, as shown in Table 2. It was observed that the remelting track in process 7# was flatter, and the remelting tracks were continuous without interruption. The PAR process was conducted as follows: the remelting current was set at 100 A, the powder feeder speed was maintained at 1 r·min^−1^, and the ion gas was 2.5 L·min^−1^. Additionally, the powder feeding gas and shielding gas flow rates were, respectively, kept at 3.5 L·min^−1^ and 12 L·min^−1^.

### 2.2. Microstructural Characterization

The metallographic specimens were cut from the surfaces of the additive manufacturing part and the PAR part. They were then etched for 10 s using a marble etchant, which consisted of anhydrous copper sulfate (10 g), concentrated hydrochloric acid (50 mL), and distilled water (50 mL). The differences in microstructure were characterized using a Nikon ECLIPSE MA200 inverted optical microscope (OM) (Nikon Corporation, Tokyo, Japan) and a field-emission scanning electron microscope (FE-SEM, Zeiss Gemini SEM 500) (Carl Zeiss AG, Oberkochen, Germany) coupled with an energy dispersive X-ray spectrometer (EDS) (Oxford Instruments, Abingdon, UK). The solidification structures were characterized with a transmission electron microscope (TEM, JEM-200CX) (JEOL Ltd., Tokyo, Japan) at an accelerating voltage of 200 kV.

### 2.3. Property Testing

The tensile strength of the surface specimens of the remelted parts and the additive manufacturing parts was tested using an INSTRON electronic tensile testing machine (Instron Corporation, Norwood, MA, USA) at room temperature to evaluate the impact of remelting on the mechanical properties of the additive manufacturing parts. The machine was accompanied by a 12.5 mm instron extensometer to accurately obtain the tensile strain of the tensile specimens. The specimens were subjected to a tensile test at a rate of 0.3 mm/min The wear resistance and corrosion resistance were evaluated using an ML-100 tribometer (CSM Instruments, Peseux, Switzerland) in a sliding wear pin-on-disk experiment with an applied load of 10N, and CHI760E series of electrochemical test stations (CH Instruments, Inc., Austin, TX, USA), respectively. Specifically, a three-electrode system was used, comprising the specimen as the working electrode, a saturated calomel as the reference electrode, a platinum electrode as the auxiliary electrode, and 3.5% NaCl solution as the corrosion environment. Eventually, the wear and corrosion morphology were characterized in detail with a scanning electron microscope (FEI-Q25, Thermo Fisher Scientific, Eindhoven, The Netherlands) and OLS4000 laser confocal microscope (LSCM, Olympus Corporation, Tokyo, Japan).

## 3. Results and Discussion

### 3.1. The Tensile Property Results

**Figure 4:**
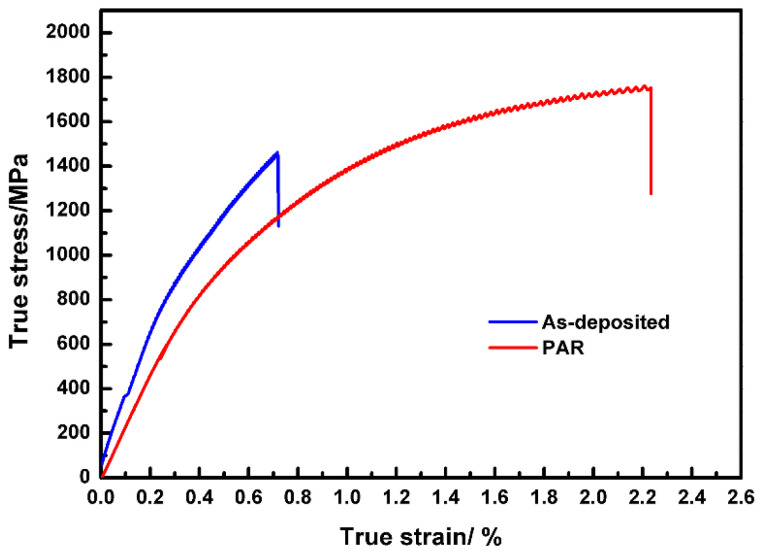
The results of the tensile properties of the as-deposited part and the remelting part.

Three replicate tensile tests revealed high consistency, and a representative curve was selected to reflect the typical mechanical response, as shown in Figure 4. Figure 4 shows the tensile property results of the surface tensile specimens before and after remelting. It can be seen that the tensile strength of the as-deposited part was 1463 MPa, and its elongation was 0.7%. After remelting treatment, the tensile strength reached 1753 MPa, and the elongation increased to 2.3%. The strength and elongation increased by 20% and 229%, respectively.

### 3.2. Wear Resistance and Corrosion Resistance

**Figure 5:**
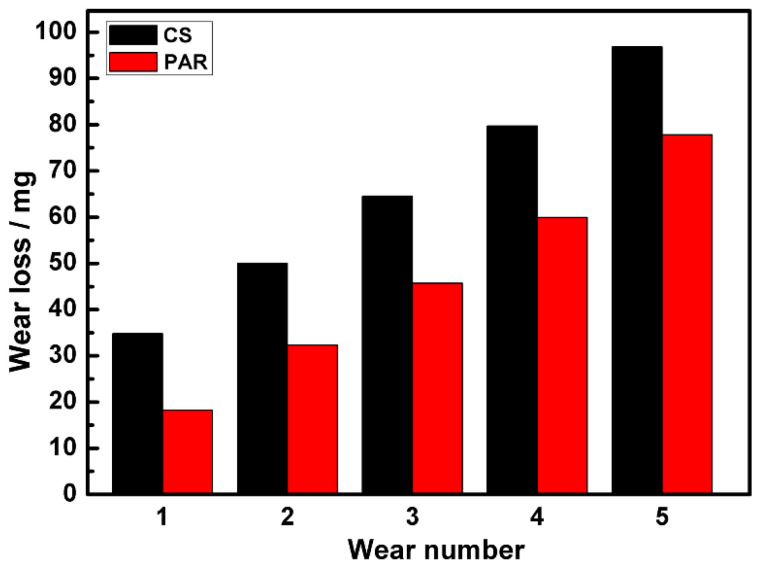
The results of the cumulative weight loss of as-deposited part and the remelted part.

Figure 5 reveals the cumulative wear losses of the as-deposited part and the PAR part. Each specimen underwent five tests. The results show that the wear loss of the as-deposited part (96.84 mg) exceeded the cumulative wear amount of the PAR part (77.79 mg), reducing the cumulative wear loss of the PAR specimen to 80% of the as-deposited part.

**Figure 6:**
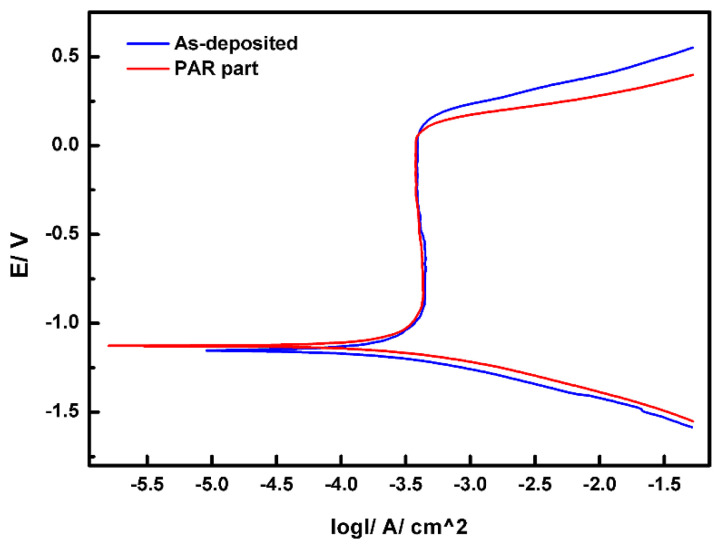
The electrochemical polarization curves of the as-deposited part and the PAR part.

Figure 6 presents the electrochemical polarization curves of the as-deposited part and the remelted part. It was observed that both steels exhibited obvious positive electrode passivation. The positive electrode passivation limited the emission of metallic ions and prevented their further corrosion to a certain extent. It was observed that the self-corrosion potential (*Ecorr*) did not experience a significant increase after the PAR treatment.

This indicates that the early passivation behavior of the steels was not sensitive to the microstructural changes caused by remelting treatment. However, the stability of the passivation film slightly declined after the remelting treatment. The passivation current density (*Icp*) values of the two types of steel were similar, ranging from −3.43 A·cm^−2^ to −3.44 A·cm^−2^. The point corrosion potential (*Ep*) of the PAR part was slightly lower than that of the as-deposited part, and its corrosion resistance was slightly reduced.

Although the differences in *Icorr* and *Ecorr* from Tafel plots are small and lack statistical validation due to limited replication, consistent trends suggest a measurable, albeit minor, effect of surface remelting on the corrosion behavior of the same steel grade, likely linked to near-surface microstructural changes.

### 3.3. Microstructure of As-Deposited and PAR Part

**Figure 7:**
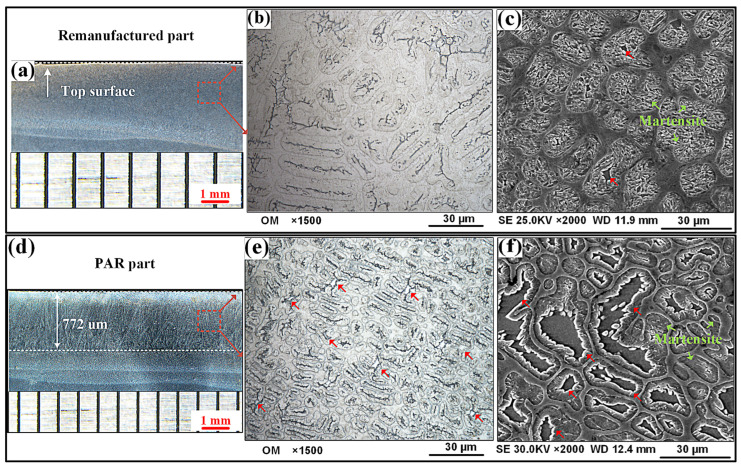
OM images of longitudinal section microstructures of (**a**) as-deposited and (**d**) PAR parts. OM images of cross-section microstructures of (**b**) as-deposited and (**e**) PAR parts. SEM images of cross-section microstructures of (**c**) as-deposited and (**f**) PAR parts.

Figure 7 depicts the microstructure modification result of the as-deposited part carried out through the PAR. Figure 7a,d illustrate the macrostructure morphologies of the longitudinal sections of the surfaces of the as-deposited part and the PAR part, respectively. Compared with the as-deposited part, the surface of the PAR part formed a 772 μm thick remelting layer (Figure 7d).

The optical microstructures of the cross section are shown in Figure 7b,d. Compared with the as-deposited part, the grain size of the PAR part decreased significantly (the average spacing of the dendrite arms was reduced from 8.76 μm to 4.45 μm). This was mainly due to the rapid cooling rate of the molten pool during the in situ PAR process. Further, the lamellar martensite structure (indicated by the green arrow) and black phase (indicated by the red arrow) were both observed within the intragranular microregion of both steels, as shown in Figure 7c,f. But the intragranular black phase became coarser like a worm-like structure after the PAR treatment, as shown in Figure 7f.

**Figure 8:**
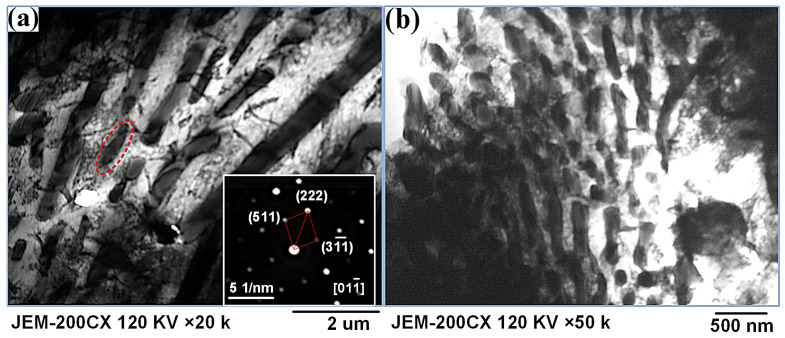
The bright-field TEM images of the intergranular region of the (**a**) as-deposited part (with the SAED pattern of its black phase) and (**b**) PAR specimen.

The intergranular structures of both steels were revealed through bright-field TEM images, as shown in Figure 8. It can be seen that whether PAR is used or not, the intergranular microstructure was characterized by the eutectic structure. The selected-area electron diffraction (SAED) pattern (inset in Figure 8a) revealed that the eutectic particle was a M_23_X_6_-type phase with a face-centered cubic (FCC) structure, where M refers to Fe and Cr, and X represents C and B. Compared with the eutectic structure of the as-deposited part, the intergranular eutectic M_23_X_6_ particles of the PAR part tended to be finer (Figure 8b).

**Figure 9:**
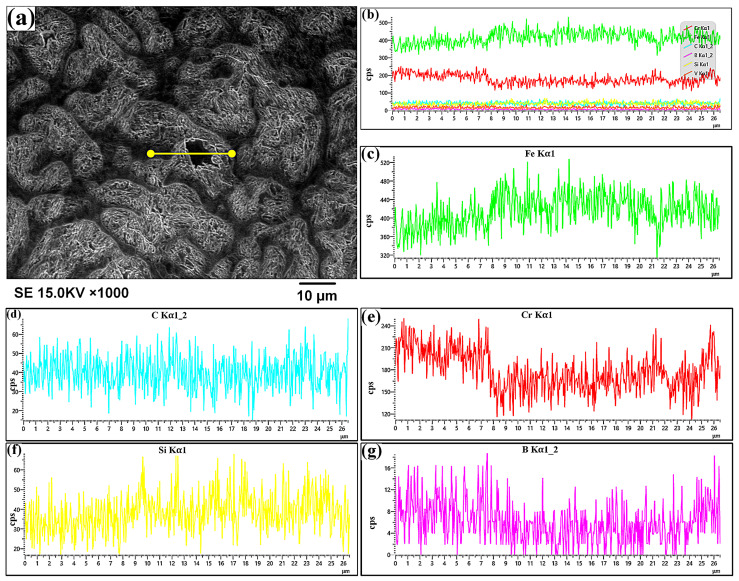
SEM-EDS results: (**a**) SEM image (region of linear scan), (**b**) EDS total spectrum, (**c**–**g**) correspond to the Fe, C, Cr, Si, and B element distributions, respectively.

Figure 9 shows the element distribution results of the intergranular structure of the as-deposited part after PAR treatment. Figure 9a,b show the region of the linear scan and EDS total spectrum, respectively. The results show that the content of Fe and Si exhibited a varying trend of increasing first and then decreasing from the intergranular region to the intragranular region and then to the intergranular area again (Figure 9c,d). The intragranular worm-like phase had the highest content of Fe and Si. The concentrations of Cr and B exhibited a trend of first decreasing and then increasing from the intergranular region to the intragranular region, and then to the intergranular area again (Figure 9c,d). The intergranular region was characterized by higher content of Cr and B.

In comparing Figure 9a,e, it was observed that the Cr content of the intragranular worm-like phase was slightly higher than that of the intragranular martensitic structure. It can be speculated that the intragranular worm-like phase was a residual high-temperature δ-ferrite phase caused by rapid solidification and cooling [28].

It can be speculated that after the remelting treatment, the increase in the tensile strength and elongation of the part is mainly attributed to the emergence of the intragranular worm-like δ-ferrite and the refinement of intergranular eutectic M_23_X_6_ particles. The research results of Zhao [29] also indicated that when the content of δ-ferrite was less than 0.4% and did not present in the form of large blocks, chains, or elongated shapes, relatively ideal impact toughness could be obtained.

The simultaneous enhancement of strength and ductility, often restricted by the strength–ductility trade-off in traditional metals, was a primary goal in advanced material processing. Recently, Zhang et al. [30] demonstrated a synergistic improvement in laser-melting-deposited 18Ni300/316 heterostructured dual-phase steel through heat treatment. The introduction of a heterogeneous lamella structure effectively overcame the strength–ductility paradox, achieving high performance via back stress strengthening. Similarly, in this study, the in situ PAR process overcame the conventional trade-off in the tailored high-strength martensitic steel. Unlike the heterostructure strengthening mechanism, the significant increase in both tensile strength (by 20%) and elongation (by 229%) was primarily attributed to unique microstructural modifications: the formation of intragranular worm-like δ-ferrite, which accommodated plastic deformation, and the refinement of intergranular eutectic M_23_X_6_ particles, which impeded crack propagation.

**Figure 10:**
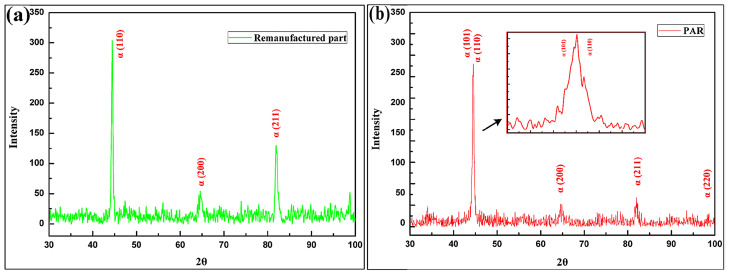
The XRD spectra results of the (**a**) as-deposited part and (**b**) PAR part.

The XRD spectra results of both steels are shown in Figure 10. The results indicate that the main phase compositions of the as-deposited part and the PAR part were essentially consistent. However, the relative intensities of the (110), (101), and (211) diffraction peaks changed after PAR treatment.

The statistical results of grain size show that the average grain size of the surface of the as-deposited part was 8.76 μm, and the average grain size of the surface of the PAR part decreased to 4.45 μm.

### 3.4. Mechanism of Wear Resistance and Corrosion Resistance

**Figure 11:**
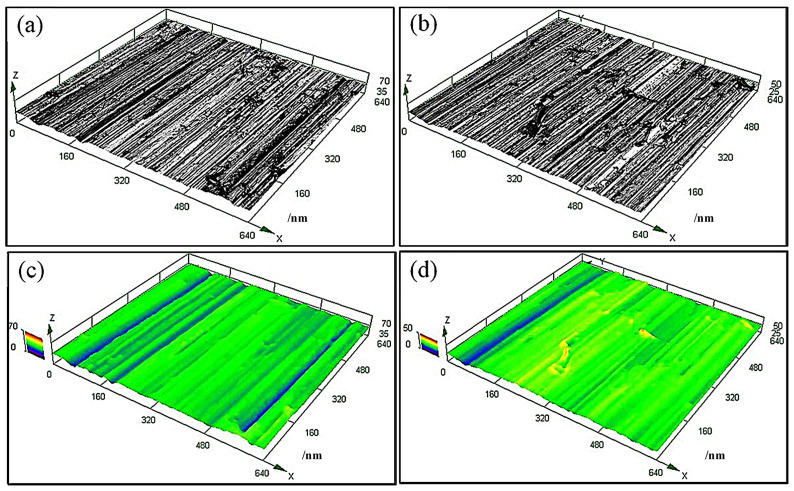
The three-dimensional wear morphology of the (**a**), (**c**) as-deposited part and (**b**), (**d**) PAR treatment.

Figure 11 shows the three-dimensional wear morphology of the as-deposited part and remelted part. By comparing Figure 11a–d, it can be observed that under the same wear test conditions, the surface wear furrows of the as-deposited part were deeper than those of the remelted part. This result indicate that the PAR part had better wear resistance than the as-deposited part.

For materials with specific components, their different wear resistance values were determined based on their microstructure. The microstructure of the as-deposited part was characterized by intragranular martensite and an intergranular eutectic structure. Additionally, intragranular worm-like δ-ferrite embedding in the martensite was clearly observed after PAR, as shown in Figure 8.

For the microstructure of the as-deposited part, lacking the worm-like δ-ferrite soft phase, fracture was the primary controlling factor for its abrasive wear. Once the crack initiated, it expanded at a very high speed, leading to cracks, fractures, and chip spalling. For the in situ PAR part, the intragranular worm-like δ-ferrite embedded in the martensite was conducive to the plastic deformation during the wear test, which led to a relatively better wear resistance compared to that of the as-deposited part.

**Figure 12:**
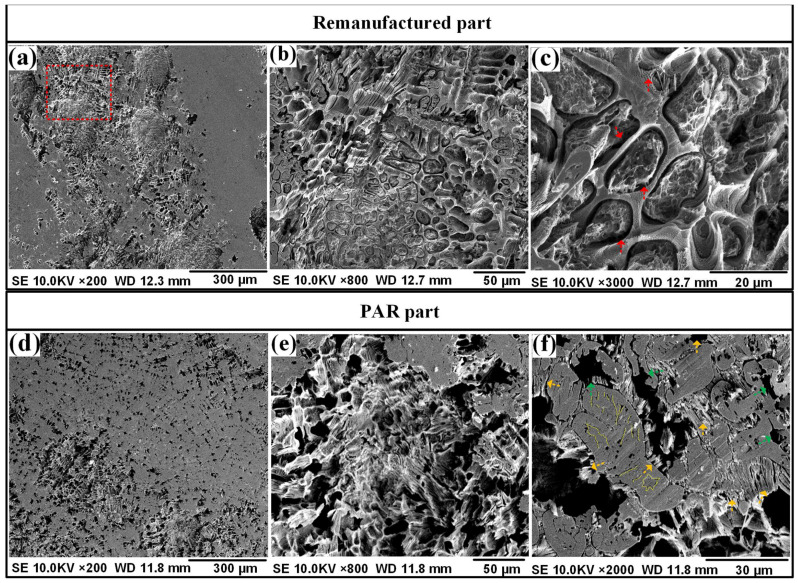
The corrosion morphologies of the (**a**–**c**) as-deposited part and (**d**–**f**) PAR part.

Figure 12 shows the corrosion morphologies of the as-deposited part and PAR part. It can be seen that intergranular corrosion was the primary corrosion mechanism of the as-deposited part, as shown in Figure 12a–c.

The same corrosion mechanism could also be observed in the PAR part, as shown in Figure 12d–f. It can be inferred that the in situ PAR process had no influence on the corrosion mechanism. This was mainly because the corrosion resistance depended largely on their microstructure of the intergranular eutectic carbides. The precipitation of intergranular carbides M_23_X_6_ caused Cr-depleted zones to form adjacent to the precipitates. The Cr-depleted zones were very anodic compared to the rest of the grains. Then, precipitations along grain boundaries created preferential paths for the anodic and cathodic reactions. This led to the intergranular corrosion of the as-deposited part and the PAR part.

## 4. Conclusions

The microstructure of high-strength martensitic steel specifically for additive manufacturing was modified using PAR treatment to improve its surface properties. The main research results are summarized as follows:

(1) The microstructure of the PAR part was intragranular martensite and an intergranular eutectic structure, and worm-like δ-ferrite could be observed in the intragranular martensite matrix. The TEM results show that the remelting treatment made the intergranular eutectic structure of the part finer after remelting treatment.
(2) Compared with the as-deposited part, the tensile strength of the PAR part reached 1753 MPa, and the ductility increased to 2.3%. The strength and elongation had increased by 20% and 229%, respectively. The two-body wear test results show that the wear amount of the PAR part had been reduced to 80% of that of the as-deposited part.
(3) The three-dimensional wear surface analysis conducted using LSCM revealed that the furrow depth profile of the wear surface of the as-deposited part was distributed within the range of 23–37 μm. The furrow depth of the wear surface of the PAR part was observed to be within the range of 16–31 μm.
(4) PAR had no influence on the corrosion mechanism of the parts. The early passivation behavior of martensitic stainless steel was not sensitive to the microstructure changes induced with PAR treatment. The passivation current density (*Icp*) values of the two types of steel were similar, ranging from −3.43 A·cm^−2^ to −3.44 A·cm^−2^.
(5) Both the as-deposited part and the PAR part exhibited significant intergranular corrosion morphological characteristics. After PAR treatment, in addition to the intergranular corrosion morphology, pitting corrosion features were exhibited. It is speculated that this is mainly related to the formation of the intragranular worm-like δ-ferrite embedded in the martensite.

## Figures and Tables

**Table 1:** The composition of Fe-Cr-C alloy (wt.%) [27].

Elements	C	Ni	Cr	Si	B	V	Fe	P	S
Composition	0.12	2.46	16.87	0.77	0.64	0.22	78.46	0.019	0.0032

**Table 2:** The remelt processing parameters.

No.	1#	2#	3#	4#	5#	6#	7#	8#	9#	10#	11#	12#
*I_r_*	170	140	100	100	100	100	100	100	100	110	120	130
*v*	5	5	5	5	5	1	1	1	1	1	1	1
*G*	2	2	2	2.5	3	2	2.5	3	3.5	3	3	3

## Data Availability

The data presented in this study are available on request from the corresponding author due to privacy restrictions.
